# The Question of Nasal Treatment for the Cure of Diseases of the Ear

**Published:** 1904-01

**Authors:** Alex. W. Stirling

**Affiliations:** M.D.; C.M. (Edin.); D.P.H. (Lond.), Atlanta, Ga.


### THE QUESTION OF NASAL TREATMENT FOR THE CURE OF DISEASES OF THE EAR.*

By ALEX. W. STIRLING, M.D.; C.M. (EDIN.); D.P.H. (LOND.),
ATLANTA, GA.

A suitable and timely subject, it appears to me, lies in the question of intranasal treatment for the relief of deafness, and in order to crystalize our ideas I shall bring forward a thesis, and put it strongly, viz.: that *intranasal treatment is ineffectual for the relief of deafness unless such treatment is also advisable for the sake of the nose itself.*

(In the term nose, in this connection, I desire to include the naso-pharynx.) With the same object in view I shall state also that those cases of deafness which are benefited by intranasal treatment are due, at least to the extent of the improvement, to Eustachian obstruction of one form or another. None of us is likely to advocate intranasal treatment for any form of external otitis, and we will all probably agree that operative shock or hemorrhage is much more likely to do harm than good in disease of the inner ear. We shall therefore dismiss diseases of the external ear and meatus, and also those of the inner ear, as they have been defined up to recent years. But I wish especially to retain for consideration among other causes of deafness what has often been called sclerosis of the middle ear, not because I consider it unrelated to the inner ear, but because so many intranasal operations have been performed with the idea of stopping the progress of the affection, one must suppose generally under the impression that it is purely a middle ear disease, and in some way amenable to such treatment. Now, all of us must have frequently met with such cases in which there was little pathological to be seen, the drum

---

*Read (by invitation) before the Southern Section of the American Laryngological, Rhinological and Otological Society, January 24, 1903.

head appearing practically normal, and movable, with a pervious Eustachian tube. We can not see the condition of the inner wall of the tympanum, and it may well be that sclerotic is replacing the normal tissue in the region of the internal foramina. I am personally inclined to agree that this disease is mainly a slow progressing change within the inner ear, and that this accounts for the poor success which up till now has attended the treatment of such cases. I would ask, upon what theory, and upon what personal experience, can one base a practice of treating the nose in order to ameliorate such a condition of things? The only justification to my mind for such treatment is to be found in those cases which, to use an Irishism, are not such cases at all, but in which there is also some middle ear affection, which has arisen by way of the Eustachian tube, that is, mixed cases, and these need not be considered separately, for any intranasal treatment would be that for the disease which complicates the sclerosis. At the same time, while this is true, it is well to bear in mind that cases seem to have been not infrequent in which the hemorrhage or the excitement frequently associated with operative measures has had a deleterious effect upon the hearing of the patients affected with sclerosis.

When we come to inflammatory states of the middle ear with the various changes in its tissue resulting therefrom, we find ourselves facing a somewhat different problem. When such cases originate in the nose, and I think they usually do, what are the actual causes and what are the steps by which they become developed and confirmed?

According to some eminent men the affection of the ear is always due to a previous affection of the Eustachian tube, a continuity of inflammation; or an easily understandable congestion of the tympanum due to a rarity of air within it, secondary to interference with its ventilation: or to a combination of the two. According to others equally eminent, a middle ear catarrh may result from a negative pressure caused by obstructions in the nose or nasopharynx or even in one nostril and acting through an open Eustachian tube.

Between these eminent men I am not going to pretend to decide and I shall merely state my belief that the negative pressure theory is still far from being proven in spite of the experiments of Dr. Scanes Spicer, who by means of pressure showed that under the circumstances in which he worked the pressure within the nose varies greatly with a varying nasal obstruction and a closed mouth. Had the mouth been open, as it is apt to be in cases of obstructed nostrils, the tracings might have been different. Wordy battles have been fought over this negative pressure theory, but it can hardly be said they have been won. We all know that complete obstruction in one or both nostrils may continue for long with no apparent effect at all upon the hearing. I have treated a number of cases of atrophic rhinitis by means of plugging the nostrils with gauze and I have never seen any ill effect accrue to the ears. Others have recorded a similar and more extended experience. It should be said, however, for the sake of justice that Dr. Scanes Spicer states that in his experiments he found that when the nostrils are totally obstructed there is not that varying pressure which accompanies a partial obstruction. And yet how often do we see a partial obstruction as by polypi, spurs and septal deflections without any affection of hearing. Do we ever find catarrhal changes in the middle ear with an entirely normal nose? Entirely normal noses are not very common, but without being able to give figures it strikes me that the kind of pathological and nasal changes which are usually associated with middle ear catarrh are found in the mucous membranes in contradistinction to bony or cartilaginous excrescences or polypi, and that these inflammatory changes of the nasal mucous membrane are to have been generally present, the kind of nasal affections which one would naturally expect not to cease at the mouth of the Eustachian tubes but to be continued along them. We best see the sort of thing I mean in acute coryza or inflammation of the adenoid tissue of the vault of the pharynx. Is acute middle ear catarrh which so frequently accompanies or follows these conditions due to negative pressure? It appears to me to be distinctly due to a continuation of the affection along the Eustachian tubes, of which there is usually ample evidence, and in my opinion the chronic forms of both are connected in the same way.

In those middle ear cases which are benefited by nasal treatment in what way does the happy result come about? When we treat the nose we aspire to do one of two things or a combination of them: To remove a mechanical obstruction or to reduce a diseased process. The evidence so far adduced to prove that the removal of a mere mechanical obstruction like a spur will improve an ear case appears to me to be insufficient. Such a sequence of events has of course been recorded, but not with sufficient exactitude. For instance, we are not at all certain that the spur was not a cause of irritation of the opposing mucous membrane, an irritation continued into the ear.

In view of the numerous cases of partial occlusion of the nostrils in which there is no affection of the ear we should be cautious in crediting the mechanical theory, because the physical laws of air pressure are constant, while the supposed results are very inconstant. On the other hand when we cure a diseased condition such as an inflamed nasal mucous membrane we remove the probable *fons et origo* of the aural affection, and at least improve a tissue directly continuous with and similar to that of the ear. We do not merely remove a partial obstruction; we substitute a healthy for an unhealthy process. That may be in front of the ear in the true nasal mucous membrane, or it may be behind it. When a boy immediately after the removal of his adenoids hears the whispered voice at twelve feet who immediately before the operation heard a similar sound at only twelve inches, is that the result of the removal of a mechanical obstruction? I think it is due to the hemorrhage which depletes the walls of the Eustachian tube and perhaps of the middle ear. That the ear cases amendable to nasal treatment are, or have been, Eustachian tube cases is borne out by the observation that such are usually worse during a coryza or are improved by inflation of the middle ear, and these two points may be used as aids in giving a prognosis. The conclusion of the matter is that nasal treatment is unprofitable to the patient in cases of pure sclerosis and that in aural catarrh it should be limited to the removal of conditions which may be interfering with the recovery of the mucous membrane lying between the nose and the tympanum. This is a very different thing from a pernicious attack upon every irregularity which one may be able to discover within the nose. Such *cacoethes operandi* has already done much to bring discredit upon nasal surgery. A good rule is the proposition with which I started, viz.: that a nasal operation for the sake of the ear should be one which is advisable for the sake of the nose. While the foregoing is, for the purposes of this paper, mainly correct, in my opinion, I would like to add that I consider that in some cases of slight adenoids situated around the orifice ot the Eustachian tubes and associated with middle ear catarrh (I do not mean sclerosis on which no such operation will have a good effect) it is advisable to remove them even when there may be few or no symptoms referable to the naso-pharynx. Of course it may be asserted that there never are cases of adenoids in which their removal is not advisable for the sake of the present or future state of the respiratory tract, but into that question there is now no necessity to enter. There are several side-issues in connection with the subject of this paper into which I have not gone at all, for instance the questions, what constitutes nasal obstruction, whether there are such conditions as purely subjective nasal obstructions, latent nasal obstruction appearing only during sleep, and so on, but this paper is not intended to be exhaustive in any respect, rather merely suggestive and I trust that by others present more light may be thrown than has emanated from the writer.

